# Effect of Non-Thermal Plasma Treatment of Contaminated Zirconia Surface on *Porphyromonas gingivalis* Adhesion and Osteoblast Viability

**DOI:** 10.3390/ma15155348

**Published:** 2022-08-03

**Authors:** Seon-Ki Lee, Min-Kyung Ji, Yu-Jin Jo, Chan Park, Hoonsung Cho, Hyun-Pil Lim

**Affiliations:** 1 Department of Prosthodontics, Daejeon Dental Hospital, Wonkwang University, Daejeon 35233, Korea; leesunki@gmail.com; 2 Dental 4D Research Center, Chonnam National University, 33 Yongbong-ro, Buk-gu, Gwangju 61186, Korea; nada-juliet@hanmail.net; 3 Department of Prosthodontics, School of Dentistry, Chonnam National University, 33 Yongbong-ro, Buk-gu, Gwangju 61186, Korea; nyota66@gmail.com (Y.-J.J.); upgradepc@hanmail.net (C.P.); 4 School of Materials Science & Engineering, Chonnam National University, 33 Yongbong-ro, Buk-gu, Gwangju 61186, Korea

**Keywords:** zirconia implant, non-thermal plasma, peri-implantitis, inhibition of biofilm formation, osteoblast viability

## Abstract

Plasma treatment on a zirconia surface prevents bacterial contamination and maintains osteoblast activity. To assess the degree of adhesion of *Porphyromonas gingivalis* on a zirconia surface after non-thermal plasma (NTP) treatment, specimens were treated with plasma for 60, 300, and 600 s, after which *P. gingivalis* was inoculated onto the surface and incubated for 48 h. To assess osteoblast activity after NTP treatment, osteoblasts (MC3T3-E1) were dispensed onto the specimens contaminated with *P. gingivalis* immediately after NTP for 60 and 120 s, followed by incubation for 48, 72, and 96 h. *P. gingivalis* was cultured after 60 s of NTP treatment of zirconia. The NTP and control groups showed no significant difference (*p* = 0.91), but adhesion was significantly increased following NTP treatment for 300 s or longer (300, 600 s groups) (*p* < 0.05). After NTP treatment of *P. gingivalis*-contaminated zirconia, osteoblast activity significantly increased at 72 and 96 h (I60 and I120 s group) in the groups treated with plasma (*p* < 0.017). Application of NTP to dental zirconia implants for 60 s not only inhibits the proliferation of *P. gingivalis*, which causes peri-implantitis but also increases osseointegration on zirconia surfaces contaminated with *P. gingivalis*.

## 1. Introduction

Titanium and its alloys are widely used in dental implants [1]. However, because of adverse immunological reactions to titanium and the cosmetic limitations of metals, there is a growing need for new implant materials [2,3,4]. Zirconia can be used in patients with titanium allergy and shows it has advantages over titanium for use in oral areas where aesthetics require the use of a material with a color similar to that of natural teeth [5,6,7]. In addition, compared to titanium implants, zirconia has excellent biocompatibility and shows a lower adhesion of bacteria, thereby reducing the risk of peri-implantitis [8,9,10,11,12].

Periodontitis-related bacteria colonize exposed implants in the oral cavity within two weeks of implantation, and the entire range of subgingival flora develops within four weeks. Unlike natural teeth, implant prostheses do not possess periodontal ligaments, and the implant surface and alveolar bone are osseointegrated. Therefore, they are vulnerable to bacterial infection, leading to a high rate of alveolar bone loss in the presence of peri-implantitis, an inflammatory condition similar to periodontitis in which biofilms are formed on implant surfaces [13,14]. A biofilm consists of several species of bacterial flora, *Porphyromonas gingivalis, Aggregatibacter actinomycetemcomitans*, and *Fusobacterium nucleatum,* known as “red complexes”, participate in late colonization [15]. Particularly, biofilms related to failed implants comprise gram-negative bacilli [16]. Among them, *P. gingivalis* is a gram-negative bacillus that influences plaque maturation and is mainly observed in well-differentiated subgingival plaques. Therefore, measuring *P. gingivalis* levels in advanced peri-implantitis can predict the prognosis of implants.

Atmospheric-pressure plasma can be used to treat peri-implantitis [17]. Non-thermal atmospheric pressure plasmas, also known as cold atmospheric plasmas or low-temperature atmospheric pressure plasmas, can be generated under atmospheric conditions and at low temperatures, facilitating their clinical application to the dental treatment area [18,19]. When plasma is applied to biomaterials, chemical changes lead to alterations in surface properties such as hydrophilicity, as the oxidized layer on the surface changes based on the chemical composition of the process gas, without changing the surface shape [20]. Yoo et al. reported changes in the shape of a colony of cultured bacteria on a plasma-applied titanium surface, with more than 80% of the cells killed [21], whereas Duske et al. [22] reported that plasma treatment increased the initial adhesion of osteoblasts and promoted bone fusion, by reducing the contact angle of the surface, which increased hydrophilicity. 

Most studies have been performed in vitro by applying plasma to titanium [17]. Studies of plasma treatment of zirconia have focused on improving physical properties such as increasing the bonding strength of restoration [23,24]. Recently, changes in biological properties during the plasma treatment of zirconia were evaluated, including studies on cell activity [25], and improving osseointegration [26,27]. However, zirconia implants, peri-implantitis associated with zirconia implants, and inhibition of advanced peri-implantitis have not been widely examined.

Here, we investigated the effect of non-thermal plasma (NTP) treatment of zirconia surfaces on *P. gingivalis* adhesion and osteoblast activity in zirconia contaminated with *P. gingivalis* to determine the duration of plasma treatment that is clinically advantageous or both bacterial adhesion and osteoblast activity.

There were two null hypotheses. The first null hypothesis was that the adhesion of *P. gingivalis* would not differ after NTP treatment of zirconia. The second null hypothesis was that osteoblast adhesion would not differ in zirconia inoculated with *P. gingivalis*, even after NTP treatment of zirconia.

## 2. Materials and Methods

### 2.1. Materials

#### 2.1.1. Samples

Fully sintered zirconia was prepared in disk-shaped specimens (diameter 15 mm, thickness 2.5 mm, Zirmon^®^, Kuwotech, Gwangju, Korea) and ground and polished with #800 SiC abrasive paper while injecting water in a polishing machine (LaboPol-5, Struers Co., Catcliffe Rotherham, UK) to give the specimens an even surface roughness. All specimens were washed for 20 min with acetone, alcohol, and distilled water using an ultrasonic cleaner. After drying at 25 °C, they were sterilized in an autoclave (HS-3460SD, Hanshin Medical Co., Incheon, Korea).

#### 2.1.2. Artificial Saliva

To recreate the oral environment, artificial saliva was prepared by adding 1% mucin (M1778, Sigma-Aldrich, St. Louis, MO, USA) to the adherence buffer, as described by Li et al. [28].

#### 2.1.3. Bacterial Culture

*Porphyromonas gingivalis* (KCOM 2804), an anaerobic, gram-negative anaerobic bacterium that causes peri-implantitis, was obtained from the Korean Collection for Oral Microbiology (Gwangju, Korea) and cultured. *P. gingivalis* strains in tryptic soy broth medium (BD Biosciences, Franklin Lakes, NJ, USA) were cultured at 37 °C in an anaerobic chamber (Forma Anaerobic System 1029; Thermo Fisher Scientific, Waltham, MA, USA). A single colony formed on the solid medium was inoculated into and cultured in a liquid medium to prepare bacteria at a concentration of 1.5 × 10^7^ colony-forming units (CFU)/mL.

#### 2.1.4. Osteoblast Culture

A mouse osteoblastic cell line (MC3T3-E1 Subclone 4, ATCC CRL2593, Rockville, MD, USA) was cultured at 37 °C in a CO_2_ incubator at 5% CO_2_ (Forma Series II 3111 Water Jacketed CO_2_ Incubator, Thermo Fisher Scientific) inα-minimum essential Medium Gibco-BRL, Grand Island, NY, USA) containing 10% fetal bovine serum and 100 U/mL of penicillin. The culture medium was changed every three days, and the MC3T3-E1 cells were subcultured until enough cells were obtained for the experiment. Cells from generations 4–7 were used in the experiment.

### 2.2. Methods

#### 2.2.1. NTP Treatment at Atmospheric Pressure

**Figure 1:**
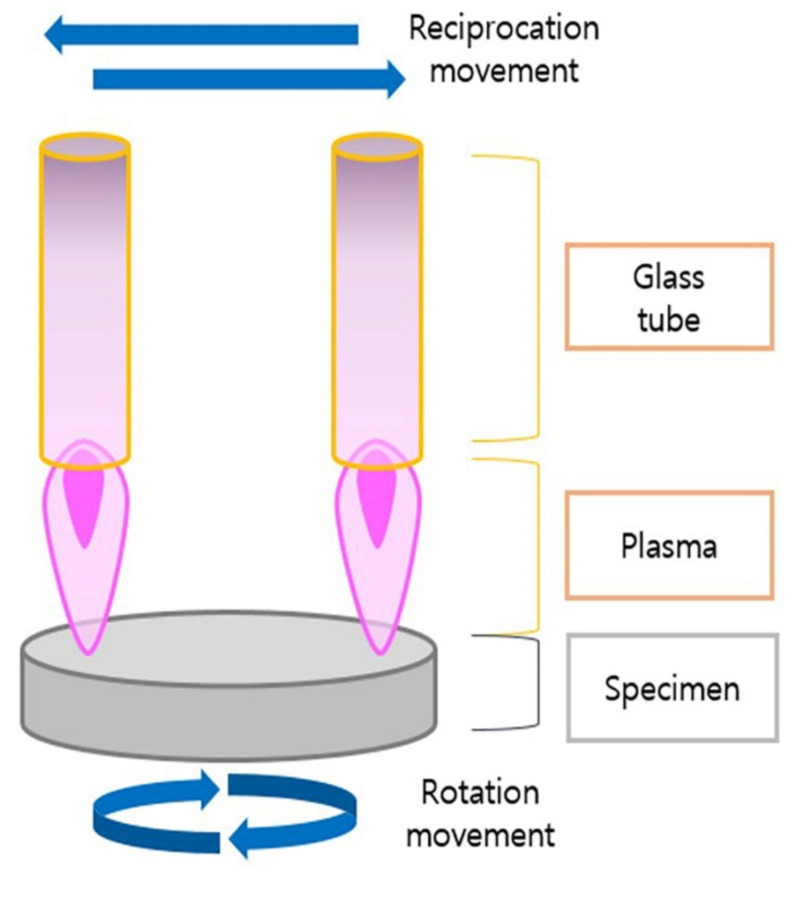
Even non-thermal plasma treatment of the specimen.

NTP at atmospheric pressure was applied to a zirconia specimen at a rate of 10 L/min and power of 300 W using a gaseous mixture containing Ar_2_ (99.2%) and O_2_ (0.8%) with an atmospheric pressure plasma generator (PGS-200, Expantech Co., Gyeonggi-do, Suwon, Korea). During plasma application, the length of the flame was maintained at 20 mm. The zirconia specimen was fixed in place with a circular jig and was rotated at 300 rpm. As the specimen rotated, the plasma alternated between left and right to ensure even application to the specimen. The reciprocation was set at 10 s per rotation and repeated automatically to match each instance of plasma application (Figure 1). 

#### 2.2.2. Assessment of Surface Characteristics

Energy-dispersive X-ray spectroscopy (EDX) (S-4700, Hitachi, Tokyo, Japan) and X-ray photoelectron spectroscopy (XPS) (VG Multilab 2000, Thermo Fisher Scientific) were performed to assess changes in the surface chemical composition of the specimens due to plasma treatment. To compare the changes in hydrophilicity, 4 µL of distilled water was dropped on the surface; after 10 s, the angle between the surface and water droplet was measured using a video contact angle meter (Phoenix 300, SEO, Kromtek, Selangor, DE, Malaysia). The average of the contact angles was calculated from the analysis of three specimens per group. Changes in the surface structure of the specimens, as well as bacterial and cell adhesion following the plasma treatment were observed using a scanning electron microscope (SEM) (S-4700, Hitachi) after coating the specimens with gold-palladium alloy using a Cressington sputter coater (108 auto, Cressington Scientific Instruments, Watford, UK).

#### 2.2.3. Assessment of Bacterial Adhesion to Specimens after Plasma Treatment 

All specimens were sterilized, and there were ten specimens in each experimental group. Bacterial adhesion to the specimen after plasma treatment was assessed in a crystal violet assay. Plasma-treated specimens were placed in a multi-well plate, and 1 mL of artificial saliva was dispensed into each well and stirred at room temperature for 2 h to coat the specimens. The artificial saliva was removed by aspiration and dried naturally for 15 min. Liquid medium (1.5 × 10^7^ CFU/mL) was dispensed into each specimen and incubated for 48 h under anaerobic conditions. The specimens were carefully washed with phosphate-buffered saline (PBS) (Welgene, Daegu, Korea) after transferring to a new well plate, to measure the amount of *P. gingivalis* attached to the specimen surface. After staining with 0.3% crystal violet solution for 10 min, the solution was removed by aspiration and washed twice with PBS. After the specimens were naturally dried for 15 min, 400 µL of destaining solution (80% ethyl alcohol + 20% acetone) was dispensed onto the specimens and decolorized for 1 h. The destaining solution (200 µL) was transferred to a 96-well plate and the absorbance was measured at 595 nm using a microplate reader (VersaMax™, Molecular Devices, Sunnyvale, CA, USA).

#### 2.2.4. Assessment of Osteoblast Activation after NTP of Specimens Contaminated with Bacteria

**Figure 2:**
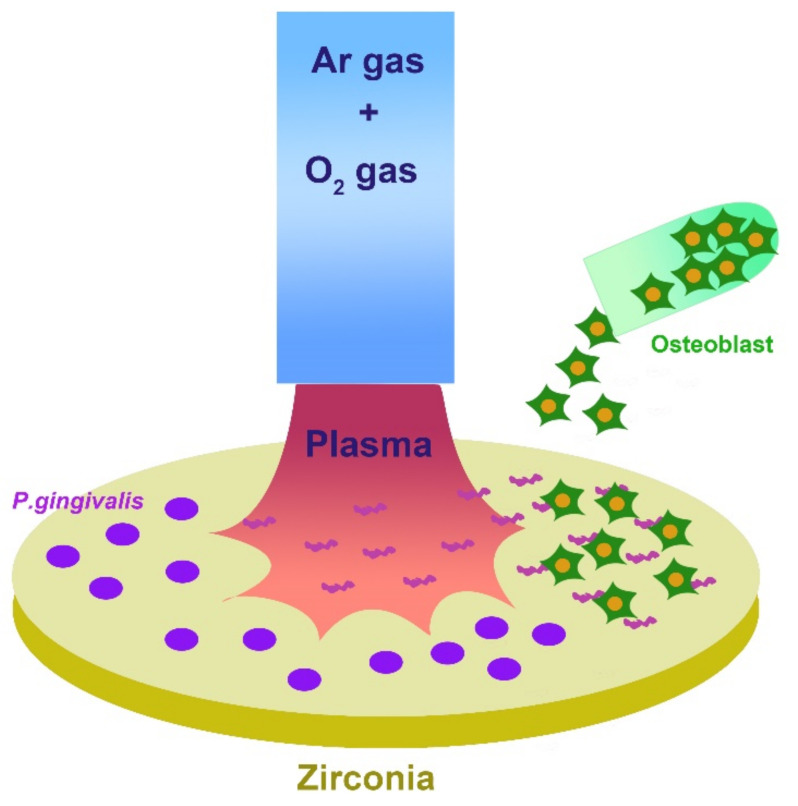
Specimens on which bacteria were cultured and treated with non-thermal plasma.

The specimens were placed in a multi-well plate, and 1 mL of artificial saliva was dispensed into each well and stirred at room temperature for 2 h to coat specimens. The artificial saliva was aspirated, and the specimens were allowed to dry naturally for 15 min. The liquid culture medium (1.5 × 10^7^ CFU/mL) was dispensed onto each specimen and incubated for 48 h under anaerobic conditions. The specimens on which the bacteria were cultured were treated with NTP for 60 s and 120 s (Figure 2). Immediately after the plasma treatment, osteoblasts (MCT3-E1; 4 × 10^4^ cells/mL) were dispensed onto the specimens and incubated in a CO_2_ incubator set at 5% CO_2_ and 37 °C. After incubation for 48, 72, and 96 h, osteoblast activation was assessed using a water-soluble tetrazolium salt (WST-8) assay. WST-8 reagent (100 μL; EZ-Cytox, Itsbio, Inc., Seoul, Korea) was dispensed into each well, and the specimens were cultured in an incubator set at 37 °C and 5% CO_2_. When the color orange developed into 100 μL of the solution was transferred from each well into a 96-well plate and absorbance was measured at 450 nm with an absorbance meter (ELISA reader: ELx 800UV, Bio-Tek Instrument., Winooski, VT, USA). The contrasting wavelength was set to 630 nm. All specimens were sterilized, and each group was divided into 30 specimens, and 10 specimens were used at each time.

#### 2.2.5. Observation of Adhesion and Morphology of *P. gingivalis* and Osteoblasts

After each incubation period, the medium was removed from each well, and bacteria and cells not adhering to the specimens were carefully washed away with PBS. To obtain SEM images, cells on the specimens were fixed with 2.5% glutaraldehyde and 2% paraformaldehyde as primary fixatives at room temperature for 2 h. After washing with PBS three times (10 min each), the cells were dehydrated in an ethanol gradient (40%, 50%, 60%, 70%, 80%, and 90%) for 5 min at each concentration, and then in 100% ethanol twice (5 min each). The specimens were immersed in hexamethyldisilazane for 15 min and dried on a clean bench for 1–2 h. the dried specimens were coated with platinum for 60 s in a vacuum using a sputter coater (E-1030, Hitachi), and cell adhesion was observed using SEM (FE-SEM S-4700, Hitachi). 

#### 2.2.6. Statistical Analysis

Statistical analysis was performed using SPSS 21.0 software (SPSS Inc., Chicago, IL, USA). The statistical significance of the crystal violet assay result after 24 h was determined by one-way analysis of variance at a significance level set at *p* < 0.05. Tukey’s test was used as a post-hoc test to evaluate the significance of differences between groups. Data from the WST-8 assay after 48, 72, and 96 h did not meet the assumption of normality. The statistical significance of WST-8 assay after 48, 72, and 96 h was tested using Kruskal–Wallis test. The significance level was set at *p* < 0.05. The Mann–Whitney test was used as a post-hoc test to determine the significance between groups, and type I errors were corrected using Bonferroni’s method. The significance level was set at *p* < 0.017.

## 3. Results

### 3.1. Surface Characteristics 

**Figure 3:**
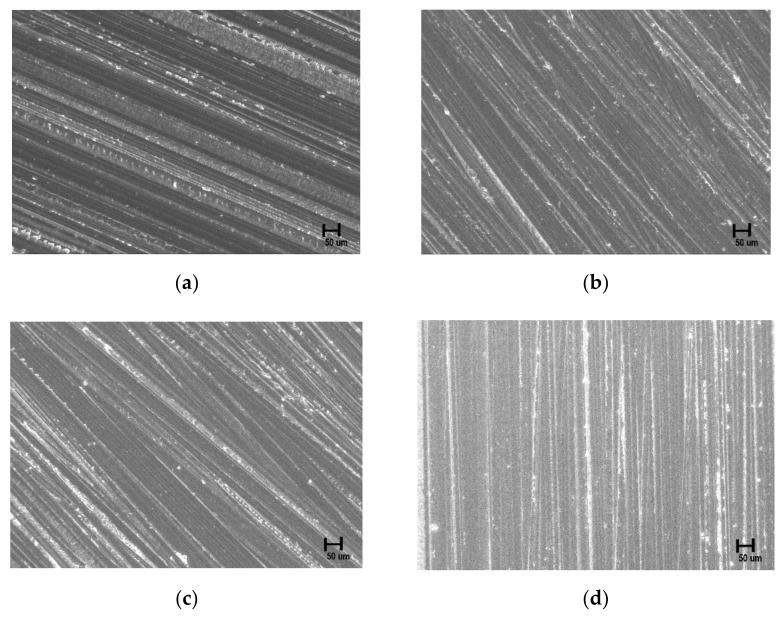
Field emission-scanning electron microscopy surface images (**a**) control group, (**b**) 60 s group, (**c**) 300 s group, (**d**) 600 s group (×3000).

**Figure 4:**
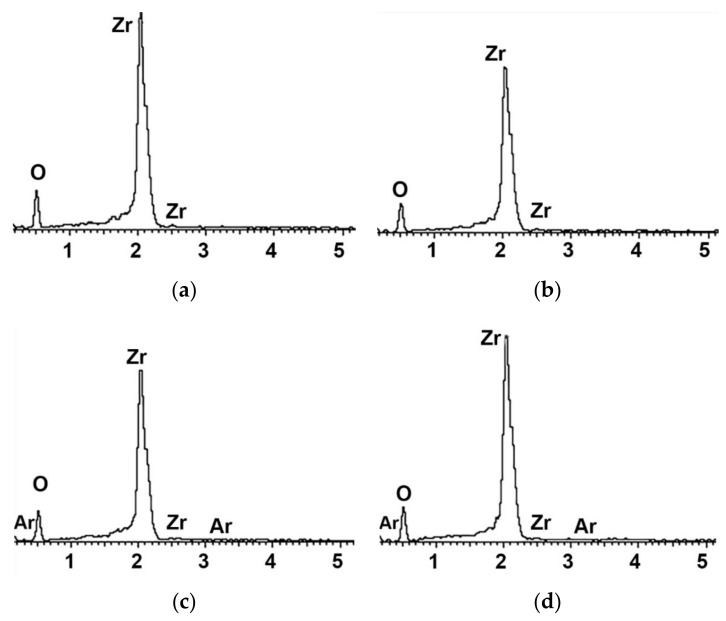
Energy-dispersive X-ray spectroscopy elemental spectrums of the (**a**) control group, (**b**) 60 s group, (**c**) 300 s group, (**d**) 600 s group.

Figure 3 shows that the structure of the zirconia surface did not differ before and after NTP treatment for the different treatment durations. The elemental content on the surface before and after plasma treatment was measured, and changes after treatment were confirmed, using EDX analysis (Figure 4). 

A microscopic amount of Ar was detected in the zirconia specimen treated with NTP for more than 300 s. The weight ratio and element ratios for each group are listed in Table 1. The carbon peak decreased in the 60, 300, and 600 s groups following NTP treatment of the zirconia surface compared to in the untreated control group, according to the XPS results. In the carbon 1 s spectrum, the peak was at 28,813.03 counts/s in the control group, whereas the peaks were 12,121.69, 9411.18, and 13,346.04 counts/s in the 60, 300, and 600 s groups, respectively (Table 2). 

**Figure 5:**
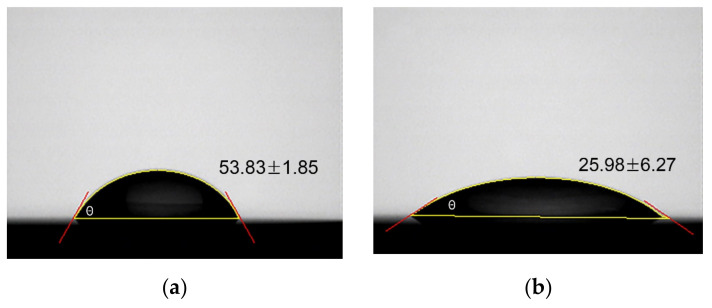

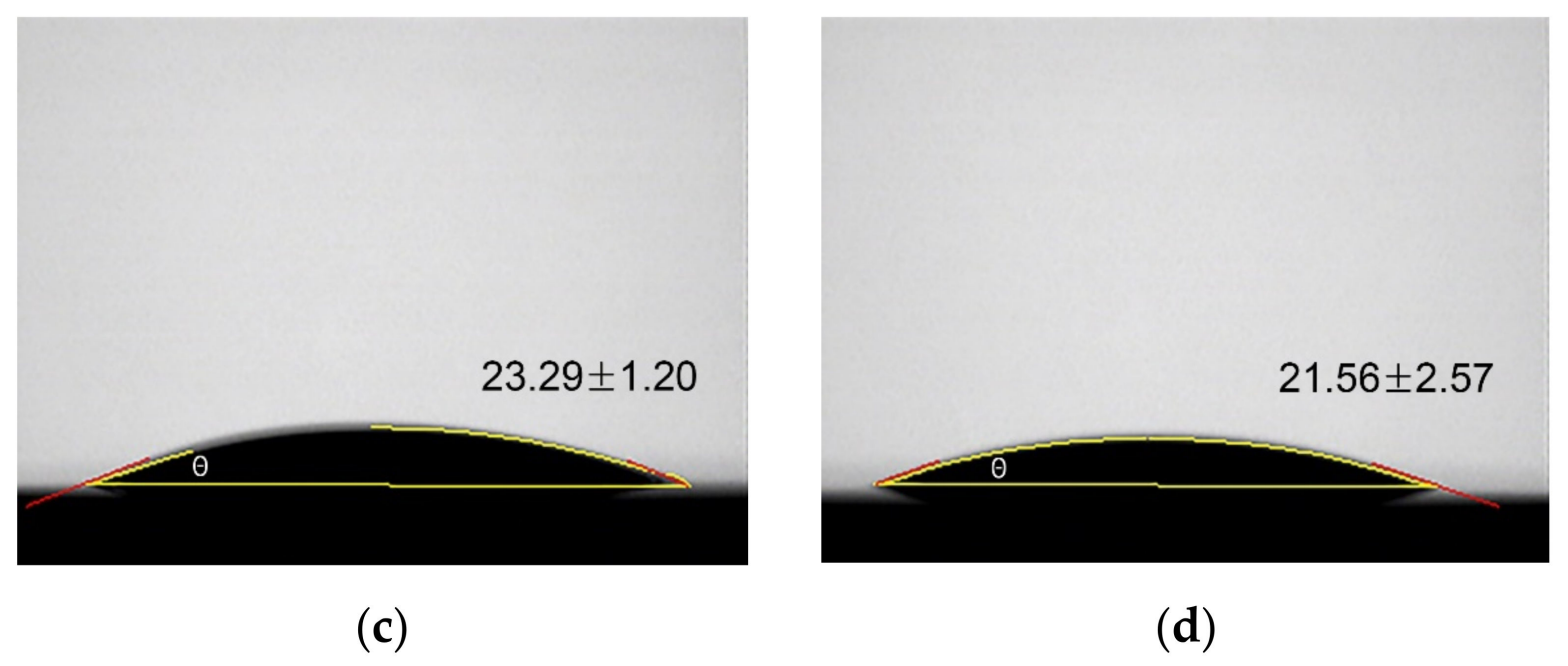
Water contact angle of specimens. (**a**) control group, (**b**) 60 s group, (**c**) 120 s group, (**d**) 180 s group.

Changes in the oxygen 1 s spectrum slightly increased when plasma was applied to the specimens for 60 s, with no significant difference when plasma was applied for 300 and 600 s. Figure 4 shows the changes in the contact angle of the zirconia surface because of NTP treatment. The contact angle decreased in the NTP-treated groups (60, 120, 180 s group) compared to that in the untreated control group. The average contact angle was 53.83° in the control group and 25.98°, 23.29°, and 21.56° in the 60, 120, and 180 s groups, respectively (Figure 5). Particularly, the contact angle spread immediately after distilled water came into contact with specimens treated with plasma for more than 300 s. The number of specimens used in the experiment was three pieces for each group.

### 3.2. Biological Response

#### 3.2.1. Bacterial Adhesion after NTP Treatment on Specimens

**Figure 6:**
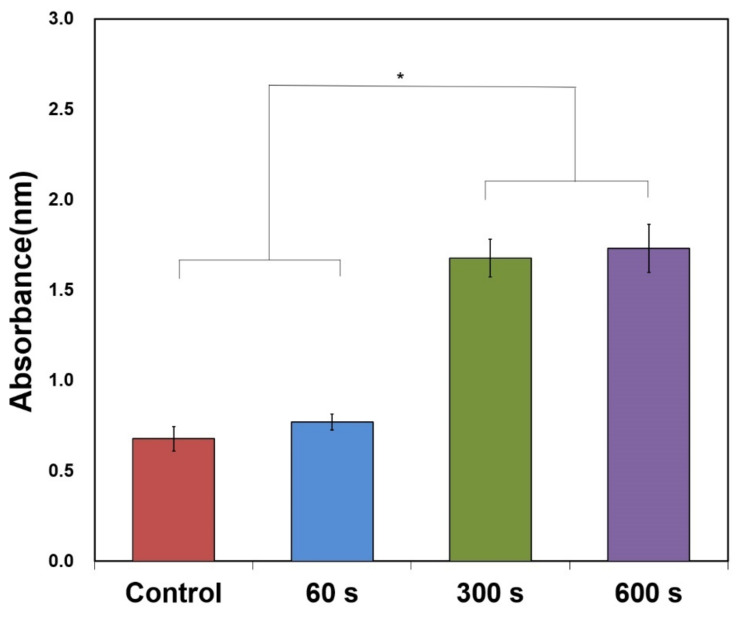
Crystal violet assay after plasma treatment. *: Significant difference at *p* < 0.05.

The group treated with NTP for 60 s showed no significant differences from the control group, whereas *P. gingivalis* adhesion was significantly increased in the groups treated with plasma for 300 and 600 s (*p* < 0.05) (Figure 6).

#### 3.2.2. Osteoblast Activation after NTP Treatment of Bacteria Contaminated Specimens

**Figure 7:**
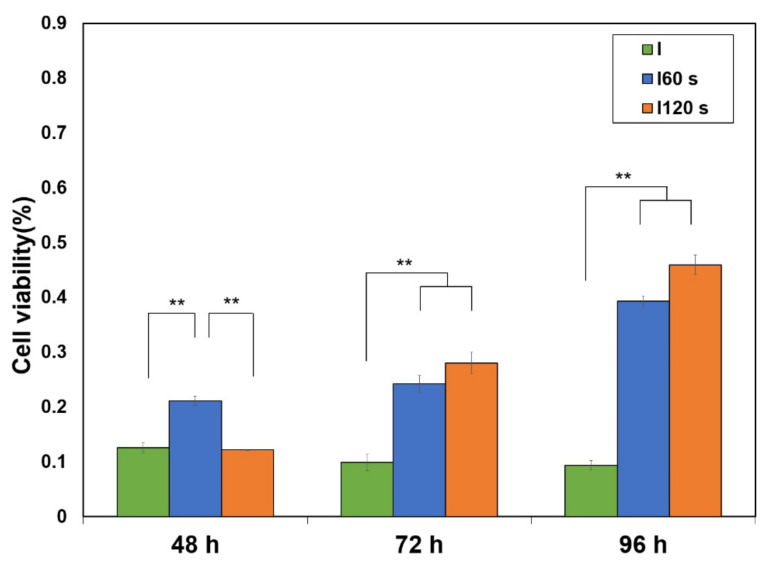
Evaluation of MC3T3-E1 cell viability. **: Significant difference at *p* < 0.017.

After 48 h, osteoblast activity was significantly increased in the group treated with plasma for 60 s; after 72 and 96 h, osteoblast activity was significantly increased in all plasma-treated groups (I60 s, I120 s) (Figure 7) (*p* < 0.017). The plasma-treated groups showed approximately two- and four-fold higher activity than the control group (I; inoculated bacteria and no NTP treatment) after 72 and 96 h, respectively.

#### 3.2.3. Microscopic Assessment of *P. gingivalis* and Osteoblast Adhesion

**Figure 8:**
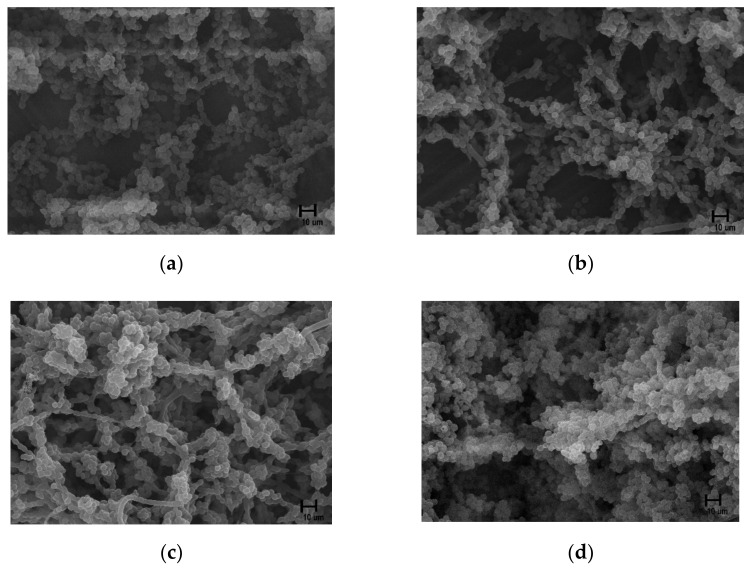
Field emission-scanning electron microscopy images of *P. gingivalis* cultured for 48 h after non-thermal plasma treatment in the (**a**) control group, (**b**) 60 s group, (**c**) 300 s group, (**d**) 600 s group (×5000).

*P. gingivalis* cultured on plasma-treated specimens was observed using SEM (Figure 8). After 48 h of bacterial culture, *P. gingivalis* was attached to the specimen in the form of bacilli in an oval shape. The shape did not significantly change according to the duration of plasma application, and the size remained similar at approximately 5 µm. *P. gingivalis* was more densely attached to the specimens of the groups treated for 300 and 600 s than to those of the control group and the group treated for 60 s. 

**Figure 9:**
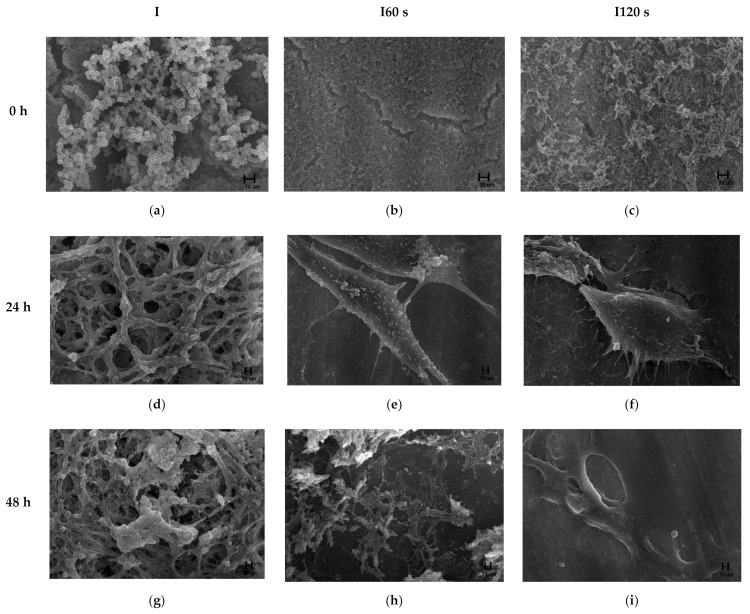

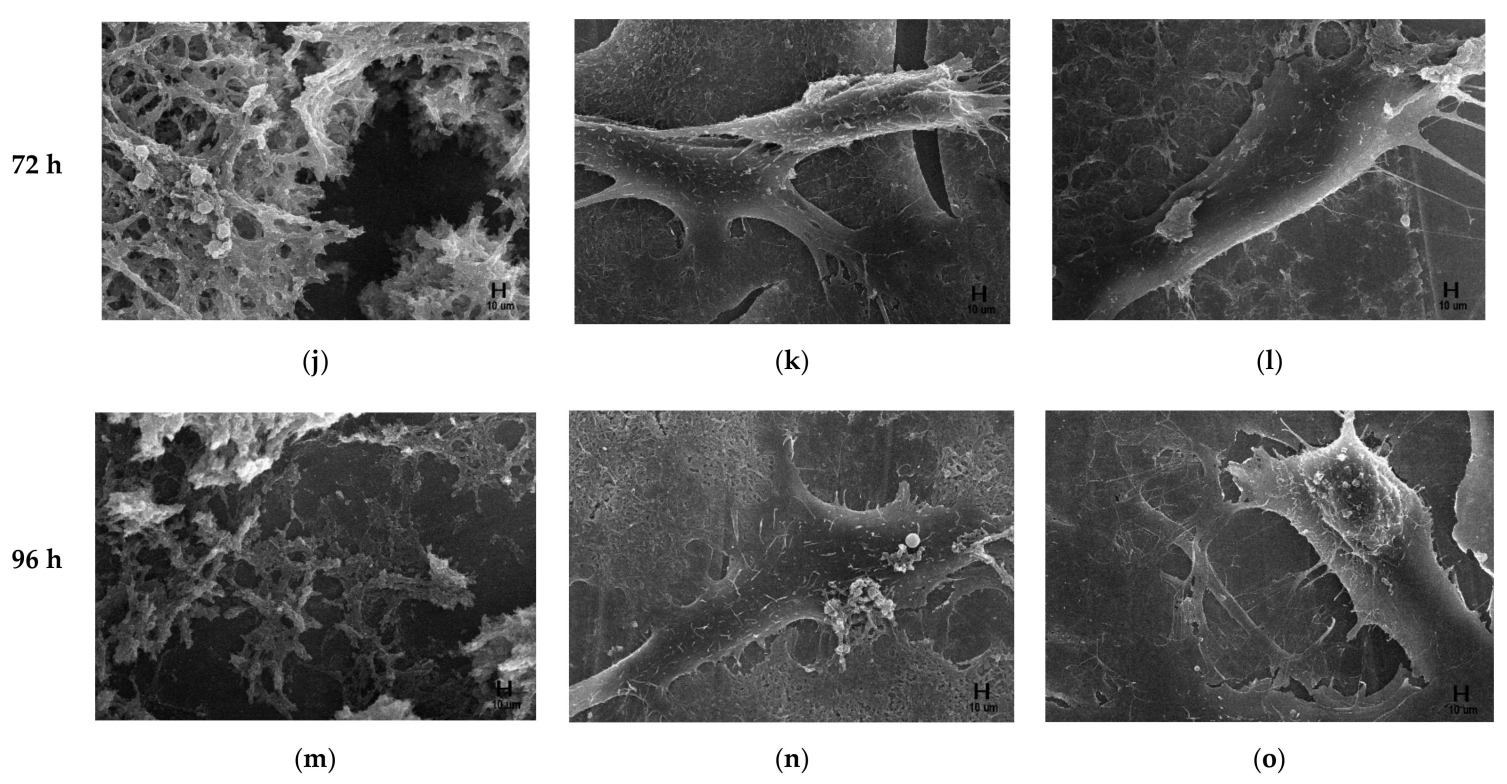
Field emission-scanning electron microscopy images of osteoblastic-like MC3T3-E1 cells cultured after 0 h (**a**–**c**) (×5000), 24 h (**d**–**f**), 48 h (**g**–**i**), 72 h (**j**–**l**), and 96 h (**m**–**o**) (×3000) on contaminated zirconia surfaces.

Zirconia specimens contaminated with *P. gingivalis* were treated with NTP, and the adhesion of osteoblasts was observed using an SEM (Figure 9). More bacteria were destroyed immediately after NTP treatment than in the control group. Additionally, bacterial destruction was more extensive in the 120 s group than in the 60 s group (Figure 9a–c). At 24 h after osteoblast injection, osteoblasts adhered in the NTP treated groups (I60, I120 s group) (Figure 9d–f). On the surface on which the dispensed osteoblasts were incubated for 48 h, osteoblast adhesion was higher in the plasma-treated groups. In the control group, bacteria and cells could not adhere to the specimens and were destroyed (Figure 9g–i). In the plasma-treated groups, osteoblast adhesion on the surface of the 72-h-incubated specimens was higher than that in the 48-h-incubated specimens, whereas, in the control group, more bacteria and cells were destroyed in the specimens incubated for 72 h than in those incubated for 48 h (Figure 9j–l). On the surface of 96 h incubated specimens, osteoblasts were abundantly distributed in the plasma-treated groups, whereas both bacteria and cells died in the control group (Figure 9m–o). Overall, as the incubation period increased, the control group exhibited the death of both osteoblast cells and bacteria, whereas, in the plasma-treated group, osteoblasts were attached to and richly distributed on the surface, and activated.

## 4. Discussion

Changes in the hydrophilicity of biomaterials promote cell adhesion and growth [25,29]. NTP treatment increases hydrophilicity by altering the chemical composition on the surface of biomaterials [30]. Duske et al. [22] reported that the adhesion and differentiation of osteoblasts showed the greatest increase when plasma was applied to a titanium surface using argon gas containing 1% oxygen as a process gas. The plasma process conditions described by Duske et al. were referenced when formulating our study; in our experiments, the shape of the zirconia surface did not change after NTP treatment, which is consistent with the results of studies in which atmospheric plasma was applied to other surfaces [31]. However, the contact angle with the surface decreased after plasma treatment compared to that on the non-plasma treated surface and further decreased with the treatment duration. We assessed the hydrophilicity of the surface and confirmed the functional groups determining the surface charge. The carbon peak on the surface of zirconia decreased with atmospheric plasma in proportion to the plasma treatment duration, and Ar was detected. Based on the detection of trace Ar when NTP was applied for more than 300 s, changes in the chemical composition of the surface may have increased when plasma was applied for more than 300 s. Previous studies [32,33] reported that after plasma application, the concentrations of CO/CO and O=CO increased and free radicals were generated by the plasma, separating the CC/CH bonds, thereby inducing the generation of OH and O groups, which are related to hydrophilicity. As carbon adversely affects cellular activity, removing carbon from the surface can enhance the adhesion, proliferation, and differentiation of osteoblasts [34,35]. In contrast, bacterial adhesion and differentiation react differently to the hydrophilicity of biomaterials. Bacterial adhesion is more favorably achieved on hydrophobic or nonpolar surfaces than on hydrophilic surfaces [36,37]. In contrast to the findings of Yoo, et al. [21], who reported that zirconia had an antibacterial effect on *Streptococcus mutans* culture after NTP treatment, we found that adhesion of *P. gingivalis* increased after plasma application. Bacterial adhesion is determined by the effects of various interactions between the bacterial film and biomaterial surface, such as hydrogen, ionic, and covalent bonding. Depending on whether the bacterial cell membrane was formed, the difference in adhesion after NTP treatment between *S. mutans* and *P. gingivalis* may be because *S. mutans* is a gram-positive coccus, and *P. gingivalis* is a gram-negative bacillus. Liu et al. [38] suggested that bacteria adhere better to hydrophobic surfaces than to hydrophilic surfaces, whereas Boks et al. [39,40] reported that *Staphylococcus epidermidis* adheres better to hydrophilic surfaces. *S. mutans* has been reported to be hydrophobic [41], and *P. gingivalis* exhibits both hydrophilic and hydrophobic characteristics [42]. Therefore, the increased hydrophilicity due to atmospheric-pressure plasma treatment on the zirconia surface decreased the adhesion of hydrophobic *S. mutans* and increased the adhesion of *P. gingivalis*. 

According to Ange et al. [43], the adhesion of bacteria increases as the roughness of the surface increases. Therefore, in this study, in order to reduce the roughness of the zirconia surface, all the specimens were finally polished for 60 s with #800 SiC grit abrasive paper after the specimen was prepared, and a smooth surface was created with the same roughness. Huh et al. [44] report the zirconia surface polished with diamond and SiC for 60 s did not cause any surface changes and was clinically acceptable. There was no change in surface roughness before and after plasma treatment, and there was no physical change in the dimensional properties of the zirconia surface before and after plasma treatment. This is in agreement with the findings of Shon et el. [27]. Additionally, Guo et el. [45] reported that plasma treatment increased osseointegration without changing the zirconia surface structure.

We investigated the activity of osteoblasts after plasma sterilization of zirconia contaminated with *P. gingivalis*. When the duration of plasma treatment was set to be identical to the duration of assessment of *P. gingivalis* adhesion activity, all *P. gingivalis* were destroyed in more than 300 s, leaving only debris; this was considered an unsuitable condition for assessing osteoblast activity after plasma sterilization treatment of zirconia contaminated with *P. gingivalis*. Therefore, considering the clinical use time, the experiment was performed for 60 and 120 s, during which not all *P. gingivalis* were destroyed. The first null hypothesis of this study (i.e., there would be no difference in the degree of adhesion of *P. gingivalis* when zirconia was treated with NTP) was thus rejected. The second null hypothesis, which was that there would be no difference in the degree of adhesion of osteoblasts even if zirconia inoculated with *P. gingivalis* was treated with NTP, was also rejected. The results of the WST-8 assay in which osteoblasts were cultured for 24 h in the specimen not treated with NTP after inoculation with *P. gingivalis* indicated that absorbance was higher than that of the plasma-treated specimens under the same conditions; this can be attributed to the fact that not only osteoblasts but also *P. gingivalis* attached to the specimens reacted with WST-8 reagent [46]. However, in specimens not treated with plasma, after 48 h, *P. gingivalis* did not increase in number and eventually died, and osteoblasts did not exhibit growth. Both the bacteria and osteoblast cells were killed at 72 h. Guo et al. [47] suggested that lipopolysaccharide, a toxic substance continuously secreted from killed bacteria, inhibits osteoblast differentiation, and induces apoptosis. Osteoblast culture after plasma treatment of specimens contaminated with bacteria has not been widely evaluated. Duske et al. [48] incubated osteoblasts obtained after plasma treatment and after physical intervention, on the surface of a titanium disk contaminated with a bacterial film, to compare the two treatment methods. Their results suggested that a combination of the two treatments is the most effective method for treating peri-implantitis. Annunziata et el. [49] reported that the argon plasma technology could be efficiently used to decontaminate/sterilize previously infected titanium implant surfaces. 

In this study, osteoblasts existed between sterilized bacterial remnants when culturing osteoblasts after only plasma treatment without physical intervention. Although not accompanied by physical procedures, osteoblast activity in the groups treated with plasma for more than 60 s increased over time compared to that in the untreated control group. Physical intervention is important in the treatment of peri-implantitis but is associated with a risk of infection and bleeding during surgical procedures. Therefore, when oral implants are exposed because of advanced peri-implantitis, plasma treatment alone can increase osteoblast adhesion and disinfection efficacy in patients who cannot undergo physical procedures because of various conditions including systemic diseases. As a limitation of this study, only *P. gingivalis* was tested among various bacteria causing peri-implantitis, and additional research on other peri-implantitis-causing bacteria is needed.

## 5. Conclusions

We investigated osteoblast activity after plasma sterilization of zirconia contaminated with *P. gingivalis* and determined the optimal plasma treatment duration for obtaining clinically favorable outcomes in terms of both bacterial contamination and osteoblast activity. We determined that although treatment with NTP for 60 s did not increase the adhesion of *P. gingivalis*, it increased the adhesion of osteoblasts and resulted in sterilization effects. However, when *P. gingivalis* was cultured after 60 s of NTP treatment of zirconia, there was no significant difference from the control group (*p* = 0.91), but the adhesion was significantly increased in the group treated with plasma for 300 s or longer (300 and 600 s groups) (*p* < 0.05). Thus, treatment with NTP for more than 60 s may lead to an increase in bacterial adhesion rather than antibacterial effects. In the treatment of inflammation around zirconia, using NTP maybe a beneficial alternative for patients who cannot be treated with physical intervention due to conditions such as systemic diseases. However, among the various bacteria that cause peri-implantitis, its use is limited to *P. gingivalis*, and additional research is needed on the treatment of inflammation and osseointegration of NTP on other dental materials such as titanium materials contaminated with other types of bacteria.

## Figures and Tables

**Table 1:** Elemental value following plasma treatment.

	Group	Control		60 s		300 s		600 s	
Element									
		Weight	Atom	Weight	Atom	Weight	Atom	Weight	Atom
O		28.58 *	69.53	27.77	68.67	26.73	67.53	26.90	67.66
Zr		71.42	30.47	72.23	31.33	73.24	32.45	72.95	32.18
Ar		-	-	-	-	0.02	0.02	0.16	0.16

*: %, sum is 100%.

**Table 2:** X-ray photoelectron spectroscopy C1s & O1s counts/s following plasma treatment.

	Group	Control	60 s	300 s	600 s
Element					
Carbon (C1s)		28,813 *	12,121	9411	13,346
Oxygen (O1s)		148,403	171,896	129,183	153,686

*: counts/s.

## Data Availability

Not applicable.
